# A Manual of Medical Diagnosis: Being an Analysis of the Signs and Symptoms of Disease

**Published:** 1862-06

**Authors:** 


A Manual of Medical Diagnosis: Being an Analysis of the Signs and Symtoms of Disease. By A. W. BARCLAY, M.D., Cantab. & Edin.; Fellow of the Royal College of Physicians; Assistant Physician to St. George's Hospital, &c., &c. Philadelphia: BLANCHARD & LEA. 1862.

This is an octavo volume of 451 pages, published in good style and on fair type. The first edition has been long enough before the profession to have its merits well known; and the early appearance of a second edition is very good evidence that its merits are appreciated. The nature and objects of the work are clearly indicated in the title. It embraces a pretty full consideration of the signs and symptoms of disease, with special reference to their bearing on diagnosis. It is a work of real value, both to the student and the practitioner. For sale by W. B. Keen & Co. of this city.

